# Next-Generation Sequencing: From Understanding Biology to Personalized Medicine

**DOI:** 10.3390/biology2010378

**Published:** 2013-03-01

**Authors:** Karen S. Frese, Hugo A. Katus, Benjamin Meder

**Affiliations:** 1 Department of Internal Medicine III, University of Heidelberg, Heidelberg 69120, Germany; E-Mail: karen.frese@med.uni-heidelberg.de; 2 DZHK (German Centre for Cardiovascular Research)—partner site, Heidelberg/Mannheim, Department of Internal Medicine III, University of Heidelberg, Im Neuenheimer Feld 350, Heidelberg 69120, Germany; E-Mail: hugo.katus@med.uni-heidelberg.de

**Keywords:** next-generation sequencing, genomics, epigenomics, transcriptomics, cardiomyopathy, heart failure

## Abstract

Within just a few years, the new methods for high-throughput next-generation sequencing have generated completely novel insights into the heritability and pathophysiology of human disease. In this review, we wish to highlight the benefits of the current state-of-the-art sequencing technologies for genetic and epigenetic research. We illustrate how these technologies help to constantly improve our understanding of genetic mechanisms in biological systems and summarize the progress made so far. This can be exemplified by the case of heritable heart muscle diseases, so-called cardiomyopathies. Here, next-generation sequencing is able to identify novel disease genes, and first clinical applications demonstrate the successful translation of this technology into personalized patient care.

## 1. From Genes, Biology and Disease

Every cell in our organism is a highly dynamic biological system that must continuously respond and adapt to multiple intrinsic and extrinsic factors. In this ever-changing system, the genome of the cell is its only constant and master plan for its behavior. Hence, if we want to understand the very complex molecular networks of cells in health and disease, we need to enlighten the secrets of the genome itself.

Genetic research has begun with fascinating investigations on simple phenotypic characteristics, with Gregor Mendel discovering the basics of inheritance in 1856 without even knowing the molecular composition of the genome. While he studied the color of pea seeds, his rules haven’t changed and are applicable to many diseases today. By now, over 4,000 Mendelian diseases have been recognized, with new ones discovered every year. Additionally, the era of genome-wide association studies has led to new paradigms, discovering the role of non-coding and intergenic variants and their contribution to highly prevalent, complex diseases [1,2,3]. It seems desirable to understand the single genetic contribution to each of these diseases to allow personalized diagnosis and therapies, a promise that was made after the first human genome was successfully sequenced and which has not been fulfilled yet. 

### Why Four Letters of Genetic Code Are So Complicated

It is evident that the genome is far more complex than previously thought. While the understanding of its coding regions has considerably advanced, 99% of the non-coding sequence is still challenging researchers from different disciplines to finally unravel all of the functions of the genome. Additionally, genetic variation across different individuals and populations is higher than estimated, and the transition from common to rare variants is fluid, making interpretation of their functional relevance difficult; structural changes, such as insertions, deletions or copy number variations, are far more frequent than previously thought. For instance, the Database of Genomic Variants (DGV) lists about 60,000 CNVs, 850 inversions and 30,000 insertion/deletions identified in healthy individuals [4].

The sequencing of the genome has laid the groundwork for many investigations that improved our knowledge on the biology and molecular principles, not only of Mendelian disorders, but of human disease in general. However, at the same time, it has raised a vast amount of new questions. For example, the completion of the human genome project revealed that there are just around 20,000 protein-coding genes, a surprisingly low number in comparison to the complexity of the human organism [4,5]. It has become apparent that a biological trait is not necessarily caused by a single gene/protein or its mutation. While rare variants are a prototype for Mendelian disorders, common variants are too frequent to be disease causing. In recent years, their disease contribution and associated biological mechanisms were successfully uncovered by genome-wide association studies. Any of the common variants alone may not affect a trait, but put together, they can add up to or result in a significant phenotypic difference. Furthermore, complex diseases are heterogeneous, often as a result of the cumulative effects of genetic and environmental influences, exerting disease susceptibility over time. Hence, the genotypic components of complex diseases are not causative, but rather mediate disease risk and further result from the cumulative effect of low penetrance variants that are frequently found in the general population, usually displaying an allele frequency >1–5%. Large-scale projects, such as ENCODE, considerably increased our knowledge on these coding and non-coding regions and the functional implications of variations of the human genome [3,5]. They take the big challenge to elucidate the functional link between associated variants and phenotypic traits and the development of methods to dissect the role of common and rare variants in aggregate. Methods, such as NGS, and projects, such as ENCODE, help shed light into these unsolved mysteries, since they investigate the whole genome, transcriptome and epigenome and, therefore, are able to provide an unbiased and comprehensive view on biological systems not available before.

## 2. Next-Generation Sequencing—Towards Understanding Biology

The advances of sequencing technologies have successfully contributed in elucidating the function of the human genome. NGS technologies have gained the capacity to sequence gigabases of DNA in a high-throughput and highly efficient manner that has not been possible using traditional Sanger sequencing. While Sanger is based on gel separation of chain-terminated fragments from enzymatic synthesis [6,7], most NGS techniques are based on locally bound nano-clusters of template DNA and incorporation of fluorescent-labeled nucleotides by DNA polymerases or ligation processes. The read lengths of current NGS approaches are relatively short, due to the small sequencing colonies and progressive signal deterioration (35–500 bp), compared to traditional sequencing (1,000–1,200 bp), which in turn is compensated by its highly-paralleled fashion. Technical and chemical refinements are used to steadily increase the read lengths [8,9], but only novel technologies, as nanopore sequencing, will be able to provide substantially longer reads.

Novel platforms of the third generation are under development, which are based on real-time sequencing of the DNA templates without prior amplification [10]. Braslavsky *et al.* introduced one of the first techniques for single-molecule sequencing [11], and fluorescence-based single-molecule sequencing methods are now available from Pacific Bioscience or Helicos. Another innovative sequencing technique is the Oxford Nanopore DNA sequencer that is free of nucleotide labeling. The technology is based on an electrical current fingerprint of each nucleotide, which is produced by the nucleotides passing through a α-hemolysin nanopore. Therefore, the nanopore is immersed in a conducting fluid, and after application of a potential voltage, an electric current, due to conduction of ions through the nanopore, can be observed [12,13,14]. These improvements and maturation of third generation sequencers will make the analysis of genetic variations in genomes more feasible in the near future.

The massive data produced by current NGS systems presents a significant challenge for data storage and analysis. A number of computational tools and databases have been newly developed to handle base calling, alignment of sequence reads to a reference, *de novo* assembly, variant detection/filtering and annotation [15,16,17,18]. This basic analysis already is demanding, but the interpretation of the large number of genetic variants is far more complex. An excellent overview of suitable software tools and databases is provided by Bao *et al*. [16,19,20,21].

So far, several diseases and syndromes have been dissected by NGS approaches. Now, the systematic detection and annotation of the complete genome, as well as correct interpretation of its variations, transcription start and polyadenylation sites, exon-intron structures, splice variants and regulatory sequences is required to advance our understanding of biology. The recently published ENCODE project helped to systematically map the regions of gene transcription, transcription factor binding and chromatin modifications, assigning functional properties to 80% of the whole genome.

## 3. Genomics

**Figure 1:**
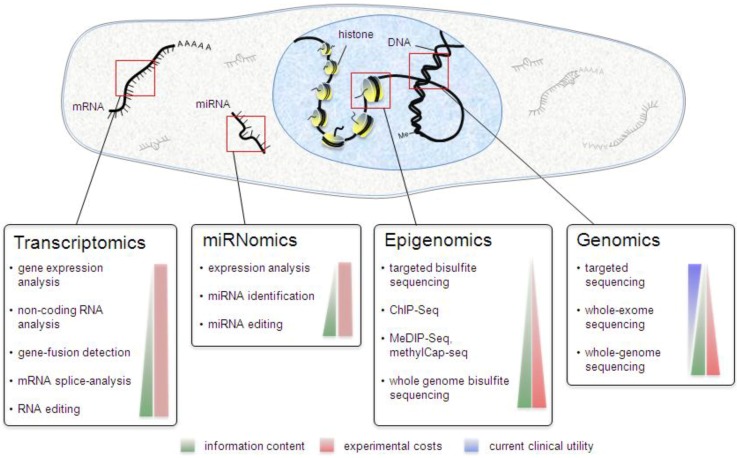
Next-generation sequencing applications. Schematogram depicting the different methods for transcriptomic, miRNomic, epigenomic and genomic studies.

In the following paragraphs, we want to provide an overview on different NGS applications, starting with genomic sequencing (Figure 1).

DNA sequencing by NGS can be applied within different applications, such as partial-exome (PES), whole-exome (WES) or whole-genome sequencing (WGS). The broad range of applications opens new and more affordable possibilities to study numerous cellular processes at the single-base resolution. However, both WES and WGS produce massive amounts of data, which presents significant challenges for data storage, distribution, analysis and interpretation. In the nearer future, this will remain one of the main bottlenecks of all described approaches.

### 3.1. Exome Sequencing

Partial and whole-exome sequencing is a relatively cost-efficient method to detect genome-wide variations in exons and adjacent splice-sites. Considering that Mendelian diseases typically affect the protein-coding regions of the genome, exome sequencing is particularly relevant to discover such rare-variants. The advances made so far in NGS technologies makes WES, with an average cost of approximately 700–1,000 US$ per sample, a widely applicable tool for discovering rare alleles underlying Mendelian phenotypes and complex traits [22,23]. To date, WES has been successfully used to identify several disease-causing genes [24], including Miller syndrome, Freeman-Sheldon syndrome [25], Floating-Harbor syndrome [26], Kabuki syndrome [27] and spino-cerebellar ataxia [28]. In the cardiovascular field, exome sequencing has successfully identified novel causal genes, including *SHROOM3* for heterotaxy [29,30] and *ANGPTL3* in cases of familial combined hypolipidemia [30,31] or novel mutations in known disease genes for dilated and hypertrophic cardiomyopathy [32,33,34]. In some cases, WES alone also fails to identify the causal variant. Galmiche *et al*. used exome sequencing in conjunction with genetic mapping to identify a mutation in the mitochondrial ribosomal protein, MRPL3, in a family with mitochondrial cardiomyopathy [35]. A combined approach of WES and copy-number variation helped Norton *et al.* to bypass missing sequencing depth to reveal a deletion in *BAG3* to be causative for familial dilated cardiomyopathy [36]. Although studies showed that WES can detect variants missed by WGS due to coverage reasons [37], it is likely that this approach will soon be superseded by high-coverage, high-quality whole-genome sequencing.

### 3.2. Whole-Genome Sequencing

Alterations in regulatory sequences and non-coding regions account for a significant proportion of genetic susceptibility to common and complex diseases. WGS holds the great advantage that it enables the grasping of variations, not only in the protein coding genes, but it also assesses the large non-coding parts of the genome. Meanwhile, WGS is only approximately five- to ten-fold more expensive than exome sequencing, and costs are expected to further decrease dramatically in the next few years [38].

Especially in cancer research, it was recognized early on that it is important to target all types of somatic/germ-line genetic alterations, including nucleotide substitution, small insertions and deletions, CNVs and chromosomal rearrangements, also of non-coding regions [39]. However, also in neurological and cardiovascular diseases, WGS is now successfully applied to dissect causative variants. Lupski *et al.*, for instance, identified by WGS a family with a recessive form of Charcot-Marie-Tooth disease, a clinically relevant heterozygous mutation in the *SH3TC2* gene [40]. Despite these achievements, the functional understanding of the millions of identified variants per genome is still challenging. Hence, integrative systems biology approaches in combination with genetic model systems, such as iPS-cells, zebrafish or mice, provide powerful tools to analyze genetic alterations and their biological effects [41,42]. Hence, only an integrative approach of *in silico*, *in vitro* and *in vivo* model systems with WGS may hold the key to facilitate the interpretation of genomic variation and allow a more accurate prediction of the clinical impact of coding, as well as non-protein-coding variations.

## 4. Transcriptomics

The DNA in multicellular organisms contains the same genetic information in every cell (with the exception of gametes or neoplastic cells); the transcriptome of different cells, however, largely varies depending on the cell type, its function and temporal state. The transcriptome describes the complete set of all RNA molecules in a cell, in the sum determined by the genes that are actively expressed and the RNAs that underlay active or passive degradation processes. The exploration of complex cellular processes, like gene expression, alternative splicing, allele-specific expression and RNA editing, are still challenging. Next-generation sequencing techniques allow analyzing RNA-levels, RNA-editing and isoform-analysis in a single, unbiased experiment. Furthermore, rRNA, tRNA and other non-coding RNAs (miRNA, ncRNA, siRNA) can be investigated.

### 4.1. Defective RNA Processing and Disease

During mRNA maturation, pre-mRNAs undergo a sequence of chemical and structural changes, such as capping, editing, splicing and polyadenylation. Alternative splicing is one main step in RNA maturation, and transcriptomic diversification is a tissue-specific and developmentally strictly regulated process [43,44]. Regulation of alternative splicing is dependent on sequence motifs in the genes to be spliced and by various splicing factors and associated proteins. Recent genome-wide analyses of alternative splicing show that at least 60% of human genes have alternatively spliced variants [45], suggesting that alternative splicing is one of the most important mechanisms to create the functional complexity of eukaryotic cells [46,47].

All of the described steps need to be well controlled [48,49,50], and hence, defects in this fine-tuned processes are increasingly recognized as probable causes of inherited human diseases [51,52,53,54,55,56]. Dysregulation of cell type-specific alternative splicing and mutations in several splicing factors have been characterized in cancer, cardiomyopathies and neurological disorders [44,57,58,59]. Currently, it is estimated that 50–60% of inherited diseases involve defective splicing, making the understanding of splicing mechanisms and regulation an important area of research [60].

### 4.2. NGS Methods to Study the Transcriptome

Until now, microarrays are still the most commonly used technique for measuring gene expression, allowing high throughput analysis of thousands of target genes in parallel. Nevertheless, microarrays have some considerable drawbacks, such as problems with unspecific hybridization and limited dynamic range. Also, they cannot be easily used to detect splice-events or previously unknown transcripts [61].

Next-generation sequencing protocols for RNAs, often referred to as RNA-seq, provide direct access to the transcript levels and sequences without the prior knowledge about the targets to be analyzed. RNA-seq is an unbiased, rapid, precise and, meanwhile, not too expensive method to quantify the expression of genes and to detect tissue-specific transcript isoforms, even without a reference genome or predesigned probes [62]. Depending on the read length, RNA-seq is able to pinpoint the location of transcription boundaries and reveals important information about how exons are connected [63,64]. Further, RNA-seq shows a high level of technical reproducibility [65], as well as a high accuracy in expression quantification [66]. In comparison to conventional microarray-based methods, it also allows the identification of sequence polymorphisms and posttranscriptional mRNA editing [62]. Further, software for *de novo* reconstruction of transcriptomes from RNA-seq data, such as Trinity, are promising approaches that allow assembly of full-length transcripts, even without a complete reference sequence [67], particularly useful for model organisms with limited knowledge about their genomes [62].

### 4.3. Gene-Expression Analyses and mRNA Splicing

In recent years, RNA-seq was successfully applied to dissect gene dysregulation that contributes to the pathogenesis of cardiovascular diseases, cancer, Chronic Obstructive Pulmonary Disease or Type 2 Diabetes, in some cases directly or indirectly caused by genetic variants in proteins involved in different steps during RNA processing [68]. Hence, variation in gene expression levels also can be hereditable [69], and polymorphisms that affect the expression levels of genes are most often found near the gene itself (cis-regulation) and especially near the transcriptional start sites [70,71,72]. Pickrell *et al.* used RNA-seq to generate a map of the transcriptional landscape of 69 lymphoblastoid cell lines derived from unrelated Nigerian individuals who had been completely genotyped by the HapMap consortium. Thus, by using this genotype data, they identified over 1,000 genes at which genetic variation influences expression levels or splicing. They also demonstrate that eQTLs near genes mostly act by a mechanism involving allele-specific expression and that variation that influences the inclusion of an exon is enriched within or near the consensus splice sites [72].

Various studies using RNA-seq have shown an improvement of assessing alternative splicing and detection of novel transcripts in comparison to splicing-arrays. RNA-seq was used on several human tissues and cell lines uncovering a larger number of alternative splicing events in humans than previously thought [43,73]. Isoforms differ most drastically between tissues, whereas differences between individuals are almost three-fold less common [74]. The dysregulation of cell-type-specific alternative splicing and mutations in several splicing factors could already be associated with various diseases [44,57,58,75,76]. It is, for instance, known that aberrant alternative splicing is tightly associated with the development of heart failure, with aberrant splicing of cardiac troponins linked to the progression of cardiomyopathies [77,78,79]. Since it is known that genetic mutations in the alternative-splicing regulators, such as RBM20 or RBM24, are associated with cardiomyopathy [80,81,82,83], RNA-seq approaches are now of major interest for target identification of these splicing factors.

### 4.4. RNA Editing and Non-Coding RNAs

Transcriptome diversity is further increased by RNA editing, which results in a different product than that encoded by the DNA template. RNA editing is a process of site-specific modification of the mRNA sequence. This process involves deamination of adenosines into inosines, which are read as guanines. The substitution of adenosine to inosine is catalyzed by members of the double-stranded RNA-specific Adenosine Deaminase enzymes (ADAR). Since RNA editing can lead to the formation of an altered protein if editing results in a codon exchange, this process may be an essential post-transcriptional mechanism for expanding the proteomic diversity [84]. Altered RNA-editing patterns were found to be associated with a number of human pathologies, including inflammation, epilepsy, depression, amyotrophic lateral sclerosis (ALS) and cancerogenesis [85,86,87,88].

For a long time, only a handful of editing sites within coding sequences have been well characterized [89]. However, this poorly understood process is becoming clearer now, due to advances in RNA-seq technologies. Bioinformatics analyses have predicted that RNA-editing is apparently more abundant than previously thought, affecting thousands of human genes [90,91]. A pioneering RNA-seq study of human brain and other tissues has revealed hundreds of new RNA editing sites, many of them located in non-coding RNAs [12].

In recent years, sequencing technologies have revealed that at least 90% of the genome is actively transcribed, giving rise to thousands of non-coding transcripts [92,93,94]. Interestingly, non-protein-coding sequences have been found to be rapidly evolving in vertebrate genomes [95] and increases proportionally with organism complexity [96], whereas the part of protein-coding genes remains relatively unchanged. ENCODE further elucidated that 80% of the genome contains functional elements defined as discrete genome segments that encode a product, for instance, protein or non-coding RNA, or display a reproducible biochemical role, e.g., transcription factor binding [1]. The ENCODE consortium mapped functional sites at high resolution across the genome integrating results from 147 different cell types and other resources, such as candidate regions from GWAS and evolutionarily constrained regions. The most prevalent functional elements identified were regions being transcribed into RNAs, including transfer RNA, microRNA, small nuclear RNA and small nucleolar RNA (tRNA, miRNA, snRNA and snoRNA). Of note, these regions covered 62% of the genome, mainly inside introns or near genes [97].

Different classes of small and large non-coding RNAs (ncRNAs) have been shown to regulate gene expression at the transcriptional level through a direct interaction with the transcriptional machinery. This results in either transcriptional activation or transcriptional repression determined by physiological and developmental processes. Some ncRNA are strongly linked to epigenetic regulation influencing chromatin-remodeling complexes, chromatin architecture, post-transcriptional processing and translation [92,98,99,100,101,102]. However, the precise functional significance of most of these non-coding transcripts remains unclear. Some of them could be considered biological noise [103], but there are already many ncRNAs that are known to have diverse functions in developmental and disease pathways [104,105,106,107,108], shown for cancer, central nervous system disorders, neurodegenerative disease and cardiovascular disease [104,109,110,111]. A recent RNA-seq analysis by Lee *et al.* revealed that more than 100 lncRNAs were differentially expressed in hypertrophic mouse hearts [112], suggesting a relevant role in proper heart function. This assumption is supported by the analysis of transcriptional levels of *ANRIL* and *MIAT* ncRNAs, which was linked to the pathogenesis of CAD and myocardial infarction [113,114,115]. Short ncRNAs (sncRNA) include miRNAs, which are the best-studied ncRNAs. They are known to be involved in the specific regulation of protein-coding genes, by post-transcriptional silencing or infrequently by activation [116,117]. Their pivotal role in several diseases has been dissected extensively in many studies about cardiovascular diseases [118,119,120], and their application as therapeutics and biomarkers is just in sight [121,122,123]. Interestingly, data from recent reports reveal that miRNAs can also regulate the expression of other types of ncRNAs (such as long ncRNAs), suggesting that miRNAs can even impact on independent regulatory networks [124,125,126,127,128,129].

## 5. Epigenomics

Transcriptional regulation is frequently controlled epigenetically by mechanisms that do not directly depend on the underlying DNA sequence [130]. Hence, to better understand the complex interactions of different regulatory factors with the genome, it is essential to perform multilayered approaches, including the analyses of gene transcription, alternative splicing, histone modification and DNA methylation.

### 5.1. Role of Epigenetic Alterations

Epigenetic modifications of the genome are now known to be involved in many cellular processes, such as embryonic development, transcription, chromatin structuring, X chromosome inactivation, genomic imprinting and chromosome stability [131]. Hence, differences in epigenetic modifications might explain changes in disease susceptibility and progression without underlying variations in the DNA sequence. Since epigenetic regulation is sensitive to environmental changes, it is thought to be a major mechanism by which external stimuli induce (an inheritable) response, together called genome-environment interaction [132].

To date, most of the epigenetic studies have focused on aberrant DNA methylation patterns, particularly in embryonic development and cancer biology [131,133,134]. Hence, methylation abnormalities were often found to occur in signaling pathways that regulate proliferation, migration, growth, differentiation, transcription and death signals. The studying of epigenetic mechanisms in different tumor types revealed that cytosine methylation is one of the earliest events in tumorigenesis. Consequently, several epigenetic markers have been identified for cancer detection, diagnosis and treatment [135], and the exploration of epigenetic alterations in other human diseases has become a special focus of current research. However, surprisingly few studies have addressed the role of epigenetic regulation in the pathogenesis of cardiovascular disease, although it is commonly recognized that not only the genetic background, but lifestyle and yet unknown factors influence cardiovascular morbidity [136,137]. This may be in part due to limited access to myocardial tissue from patients. A recent report profiled for epigenetic modifications in explanted hearts of end-stage heart failure patients [138]. This study from Movassagh *et al.* found different methylation patterns between end-stage heart failure and control human hearts in CGIs within gene promoters and gene bodies. Moreover, the observed decreased gene promoter methylation that correlated with upregulated transcripts, but not *vice versa*. By a genome-wide approach, we could recently identify alterations in cardiac DNA methylation that are associated with human dilated cardiomyopathy. By consecutive fine-mapping and biological validation in independent cohorts and *in vivo* characterization in zebrafish, it was underlined in this study that epigenetic modifications of distinct pathways and modifiers are functionally involved in the pathogenesis of DCM [139].

A recently published review by Leung *et al.* comprehensively summarizes studies that demonstrated that single nucleotide polymorphisms are assumed to be associated with altered DNA methylation and chromatin accessibility, implicating that genomic variants can modify epigenetic patterns [140,141]. Hence, the integration of DNA methylation information in current population-based studies might help to explain the impact of variants or disease-causing alleles to the onset and progression of common and complex diseases.

Unlike genome-wide variation data, which is included and steadily updated in a wealth of databases, such as the Human Genome Project, 1,000 Genomes Project or HapMap, whole-epigenome data only started to be systematically identified and catalogue. The now increasing amount of methylation data for many tissues, pathological conditions and species are deposited, for instance, in the NGSmethDB [142] or ENCODE [143] databases that will be useful for multilayered analytical approaches to improve our knowledge about epigenetic mechanisms.

### 5.2. NGS Methods to Assess the Epigenome

Bisulfite sequencing or MeDIP-Seq (methylated DNA immunoprecipitation coupled with sequencing) are used to capture global DNA methylation changes, while ChIP-Seq is applied to identify histone modifications and to analyze transcription factor binding sites [144,145]. So far, the “gold standard” for detection of cytosine methylation comprises a sodium bisulfite conversion of the DNA, followed by a sequencing step. Cytosine methylation, which is the addition of a methyl group at the carbon 5 position of cytosine through DNA methyltransferase enzymes (DNMT), plays an important role in transcriptional regulation and is the most extensively studied epigenetic modification. Recently, various high-throughput NGS approaches have been combined with bisulfite DNA conversion for genome-wide analysis of DNA methylation by discriminating methylated and unmethylated cytosines [146,147,148,149]. Alternative methods include immunoprecipitation of methylated DNA (MeDIP) [150] or Methyl-Capture sequencing (MethylCap-seq). Yu *et al.* demonstrated, for instance, the applicability of MethylCap-seq, which combines precipitation of methylated DNA by the recombinant methyl-CpG binding domain of MBD2 protein with NGS, to dissect genome-wide DNA methylation profiles of the Cisplatin-sensitive ovarian cancer cell line [151]. MeDIP has been widely used to explore the methylomes of plants, mice and human cells [152,153,154,155,156,157], and a recently improved protocol enables MeDIP-seq analysis with very low DNA concentrations [158].

Histone modifications, chromatin-remodeling factors and binding sites for transcription factors are today mostly analyzed using chromatin immunoprecipitation sequencing in combination with NGS (ChIP-seq). As compared to previously used ChIP-chip approaches, which are also based on chromatin immunoprecipitation, but on a microarray platform [159,160], ChIP-seq, on the one hand, does not have the typical microarray-specific limitations and, on the other hand, offers the opportunity for *de novo* motif discovery. Robertson *et al.* and Euskirchen *et al.*, for instance, catalogued binding sites of the transcription factors STAT1 and NRSF in human cells by ChIP-seq and highlighted the excellent resolution and low necessity of extensive replicates for the method [161,162].

## 6. NGS—Towards Personalized Medicine

The introduction of NGS technologies has tremendously changed the landscape of genomic research. As described above, NGS has led to important discoveries in biomedical research and has already been implemented in clinical diagnostics, too. Early studies reported such a successful translation of NGS into clinical workflows using whole-genome, whole-exome and enrichment-based sequencing approaches for different diseases. Especially in oncology, NGS-based diagnostic testing has already achieved a broader clinical impact. Walsh *et al*., for instance, demonstrated the clinical applicability of NGS in cancer diagnostics using target region capture and NGS to detect germ-line mutations in 21 tumor suppressor genes in genomic DNA from women with primary ovarian, peritoneal or fallopian tube carcinoma. Other publications analyzed the versatility of NGS for clinical applications for diseases, such as retinitis pigmentosa, inflammatory bowel disease, neurofibromatosis, Charcot-Marie-tooth neuropathy, Kabuki syndrome and others [40,163,164,165,166]. As recently published, we could demonstrate the diagnostic capabilities of NGS as a clinical diagnostic tool for dilated and hypertrophic cardiomyopathy [32]. Importantly, the enrichment-driven NGS approach in this study yielded consistently high sequence and target coverage, as well as good specificity and sensitivity compared to Sanger sequencing. Considering that costs of high-coverage WGS are still substantial, targeted sequencing approaches are currently delivering the best data quality for clinical applications in which a known panel of genes needs to be tested. 

In biomarker discovery, NGS is already applied in many screening studies, e.g., for miRNA or epigenomic signatures. It is foreseeable that NGS technologies can also be applied in clinical biomarker assays, especially when complex, maybe temporal or multivariate biomarker signatures are used to increase diagnostic performance [167]. At the same time, it must be noted that the benefits of NGS technologies still brings with it a number of other challenges that must be meticulously addressed before they can be transferred from the research field into routine clinical application. In particular, a comprehensive and transparent analysis strategy of the large amount of sequence information and their interpretation is indispensable. This poses a challenge to both laboratory and clinical geneticists and requires appropriate training of different disciplines, which may directly facilitate the application of genome-based medicine. Some clinical pilot studies relying on NGS in daily practice underscore the value of such a multidisciplinary team dedicated to the collection and interpretation of NGS data and establishment of “best practices” for clinical NGS [168]. Finally, we are facing numerous political, ethical and social challenges by the advancements and progression of translational genomics, bearing fears of discrimination, breach of confidentiality and data security [169,170,171]. It is in our hands to carefully address these issues and establish NGS in the clinics for the sake of our patients.
